# Intestinal absorption and biological effects of orally administered amorphous silica particles

**DOI:** 10.1186/1556-276X-9-532

**Published:** 2014-09-26

**Authors:** Tokuyuki Yoshida, Yasuo Yoshioka, Hideki Takahashi, Kazuki Misato, Takahide Mori, Toshiro Hirai, Kazuya Nagano, Yasuhiro Abe, Yohei Mukai, Haruhiko Kamada, Shin-ichi Tsunoda, Hiromi Nabeshi, Tomoaki Yoshikawa, Kazuma Higashisaka, Yasuo Tsutsumi

**Affiliations:** 1 Laboratory of Toxicology and Safety Science, Graduate School of Pharmaceutical Sciences, Osaka University, 1-6 Yamadaoka, Suita, Osaka 565-0871, Japan; 2 Laboratory of Biopharmaceutical Research, National Institute of Biomedical Innovation, 7-6-8 Asagi Saito, Ibaraki, Osaka 567-0085, Japan; 3 Cancer Biology Research Center, Sanford Research/USD, 2301 E. 60th Street N, Sioux Falls, SD 57104, USA; 4 The Center for Advanced Medical Engineering and Informatics, Osaka University, 1-6 Yamadaoka, Suita, Osaka 565-0871, Japan; 5 Division of Foods, National Institute of Health Sciences, 1-18-1 Kamiyoga, Setagaya-ku, Tokyo 158-8501, Japan

**Keywords:** Biological effects, Everted gut sac method, Intestinal absorption, Silica nanoparticles

## Abstract

Although amorphous silica nanoparticles are widely used in the production of food products (e.g., as anticaking agents), there is little information available about their absorption and biological effects after oral exposure. Here, we examined the in vitro intestinal absorption and in vivo biological effects in mice of orally administered amorphous silica particles with diameters of 70, 300, and 1,000 nm (nSP70, mSP300, and mSP1000, respectively) and of nSP70 that had been surface-modified with carboxyl or amine groups (nSP70-C and nSP70-N, respectively). Analysis of intestinal absorption by means of the everted gut sac method combined with an inductively coupled plasma optical emission spectrometer showed that the intestinal absorption of nSP70-C was significantly greater than that of nSP70. The absorption of nSP70-N tended to be greater than that of nSP70; however, the results were not statistically significant. Our results indicate that silica nanoparticles can be absorbed through the intestine and that particle diameter and surface properties are major determinants of the degree of absorption. We also examined the biological effects of the silica particles after 28-day oral exposure in mice. Hematological, histopathological, and biochemical analyses showed no significant differences between control mice and mice treated with the silica particles, suggesting that the silica nanoparticles evaluated in this study are safe for use in food production.

## Background

Nanomaterials are currently used in a variety of applications, including the production of food products. For example, amorphous silica nanoparticles are widely used as stabilizers, anticaking agents, and carriers for fragrances and flavors [1-3]. With the growing commercialization of nanomaterial-derived food additives, the opportunities for human oral exposure to nanomaterials are substantially increasing; however, there have been few studies conducted, examining the safety of nanomaterials in food products. Therefore, assessments of the absorption and biological effects of nanomaterials after oral exposure are urgently needed.

The information currently available about the biological effects and absorption of nanomaterials after oral exposure is limited. For example, 56-nm silver nanoparticles have been found to be distributed in rat liver, kidney, brain, lung, and blood after 90 days of oral exposure at doses of 30, 125, or 500 mg/kg, and a no-observed-adverse-effect level (NOAEL) of 30 mg/kg and a lowest-observed-adverse-effect level (LOAEL) of 125 mg/kg have been suggested for these nanoparticles [4]. However, there is little information about the intestinal absorption and biological effects of silica nanoparticles after oral exposure. Furthermore, an efficient method to determine the absorption of silica particles in the human body is yet to be established. Therefore, to evaluate the safety of silica nanoparticles after oral exposure, practical in vitro methods that allow prediction of the in vivo intestinal absorption and biological effects of silica nanoparticles after long-term oral exposure are urgently needed.

The everted gut sac method is a useful tool for modeling the in vitro absorption and intestinal metabolism of drugs. This method, which was first described by Wilson and Wiseman in 1954 and then later improved by Barthe et al., has been extensively used to investigate the pharmacokinetics of drug absorption, drug metabolism, and prodrug conversion in gastrointestinal segments [5-7]. This method has also recently been used to obtain information about the absorption of drugs contained in nanocarriers such as liposomes [8]. The results of these studies suggest that this method could be used to accurately measure the intestinal absorption of silica nanoparticles.

In the present study, we used the everted gut sac method to evaluate the intestinal absorption of amorphous silica particles of various diameters. In addition, we investigated the in vivo biological effects of 28-day oral administration of the amorphous silica particles in mice.

## Methods

### Silica particles

We evaluated the following particles purchased from Micromod Partikeltechnologie, Rostock/Warnemünde, Germany: amorphous silica nanoparticles (nSP) with a diameter of 70 nm (nSP70), microsilica particles (mSP) with diameters of 300 or 1,000 nm (mSP300 and mSP1000, respectively), and nSP70 that had been surface-modified with carboxyl or amine groups (nSP70-C and nSP70-N, respectively). All the silica particles were sonicated for 5 min and vortexed for 1 min prior to use.

### Animals

BALB/c mice (female, 6 weeks) were purchased from Japan SLC (Shizuoka, Japan) and Wister rats (male, 8 weeks) were purchased from Shimizu Laboratory Supplies Co (Kyoto, Japan). The animals were housed separately in a ventilated animal room that was maintained at 20°C ± 2°C under a 12-h light/12-h dark cycle. All the animal experiments in this study were performed in accordance with the National Institute of Biomedical Innovation and the Osaka University Guidelines for the Welfare of Animals.

### Everted gut sac analysis

Wistar rats (male, 8 weeks) were fasted for 12 h (ad libitum access to water) prior to the experiment. The rats were anesthetized with pentobarbital, subjected to abdominal section, and exsanguinated by transection of the descending aorta. The whole small intestine was isolated and gently flushed with Tyrode’s buffer (NaCl, 137 mM; KCl, 5.4 mM; NaH_2_PO_4_, 0.16 mM; MgCl_2_, 0.5 mM; CaCl_2_, 1.8 mM; HEPES, 5 mM; pH 7.4). A 3- to 4-cm segment of the small intestine was removed and everted over a silicone tube. The bottom portion was tied with thread and the segment was filled with 0.6 to 0.8 mL of Tyrode’s buffer. The filled segment was then placed in 2.5 mL of Tyrode’s buffer only (control group) or a solution of silica particles (12.5 mg/mL) in Tyrode’s buffer or/and incubated at 37°C for 45 min. After incubation, the solution on the serosal side of the segment was collected. An inductively coupled plasma optical emission spectrometer (ICP-OES; 735-ES, Agilent Technologies, Tokyo, Japan) was used to determine the silicon content in the solution.

### Oral exposure of mice to silica particles

BALB/c mice (female, 6 weeks) were orally exposed to nSP70, mSP300, mSP1000, nSP70-C, nSP70-N, or water (control group) at a daily dose of 2.5 mg/mouse administered by means of oral gavage for 28 days. The mice were weighed on days 7, 14, 21, and 28 of the study period.

### Blood biomarker assay

Twenty-four hours after the final oral administration of the silica particles, a blood sample was collected from the heart of each mouse by means of a plastic syringe containing 5 IU/mL of heparin sodium. Plasma was harvested by centrifuging the collected blood at 1,750 × *g* for 15 min. The plasma levels of alanine aminotransferase (ALT) and blood urea nitrogen (BUN) were determined with a biochemical auto-analyzer (Fuji Dri-Chem 7000, Fujifilm, Tokyo, Japan).

### Histopathological examination

Twenty-four hours after the final oral administration of the silica particles, the liver, kidney, brain, lung, spleen, heart, stomach, small intestine, or large intestine of the mice were excised and fixed immediately in 4% paraformaldehyde. The tissues were embedded in paraffin blocks and sectioned, and the sections were mounted on glass slides.

### Hematological analysis

Twenty-four hours after the final oral administration of the silica particles, a blood sample was collected from the heart of each mouse by means of a plastic syringe containing 0.1 mM EDTA, and the numbers of total white blood cells, lymphocytes, monocytes, red blood cells, granulocytes, and platelets in whole blood were determined with an auto-analyzer (VetScan HMII Hematology System, Abaxis, Sunnyvale, CA, USA).

### Statistical analysis

Differences between the treated groups and the control groups were compared with Tukey’s test after analysis of variance.

## Results

### Physicochemical properties of silica particles

Previously, we confirmed by means of dynamic laser scatter analysis that the mean secondary particle diameters of nSP70, mSP300, mSP1000, nSP70-C, and nSP70-N are 65, 322, 1,140, 70, and 72 nm, respectively [9]. Transmission electron microscopy revealed that the silica particles are well-dispersed smooth-surfaced spheres, indicating that they remain stable and well dispersed in solution and do not aggregate [9].

### Intestinal absorption of silica particles

**Figure 1:**
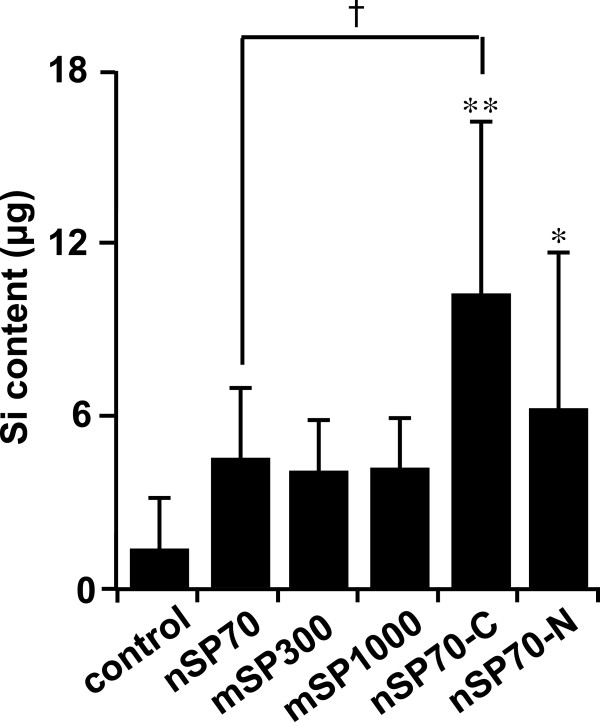
**Measurement of the absorption of silica particles using everted gut sac method.** Measurement of the absorption of silica particles in rat intestine by means of the everted gut sac method combined with an inductively coupled plasma optical emission spectrometer. Intestinal sacs were incubated in solutions of the indicated silica particles (12.5 mg/mL) for 45 min. Values are expressed as mean ± SD (*n* = 5 to 12). *P < 0.01 and **P < 0.05 compared with the control group.

We used the everted gut sac method to evaluate the intestinal absorption of silica particles. Tissue viability is a limiting factor in everted gut sac analysis; however, the viability and metabolic activity of intestinal tissue has been reported as being retained for approximately 2 h under physiological conditions [10]. Everted gut sac analysis revealed that the absorption of nSP70-C and nSP70-N from the mucosal side to the serosal side of the sacs was significantly greater than the absorption of the other silica particles after incubation for 45 min. And the absorption of nSP70-C was significantly greater than that of nSP70 (Figure 1). This suggested that silica nanoparticles are absorbed through the intestine and that the surface property of the particles is one of a determinant of the degree of absorption.

### Biological effects of silica particles

**Figure 2:**
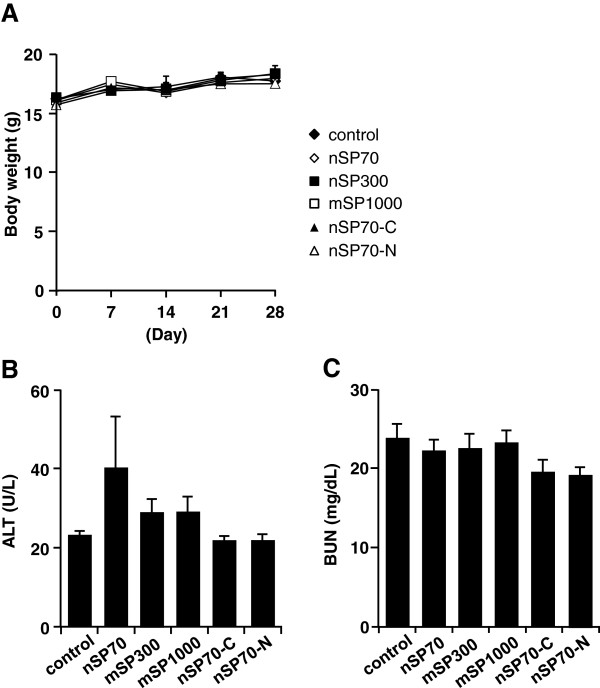
**Body weight, histopathological, and biochemical analyses after 28-day oral administration of the indicated silica particles. (A)** Body weight during 28-day oral administration of silica particles; plasma concentrations of **(B)** alanine aminotransferase (ALT) and **(C)** blood urea nitrogen (BUN). Values are expressed as mean ± SE (*n* = 4 to 11).

**Figure 3:**
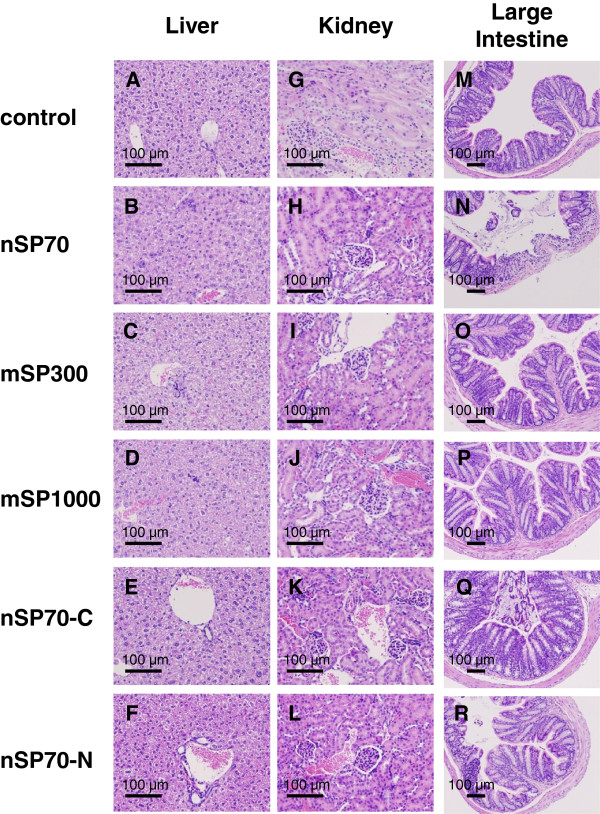
**Histopathological analysis after 28-day oral administration of the indicated silica particles. (A to F)** liver, **(G to L)** kidney, **(M to R) **large intestine.

**Figure 4:**
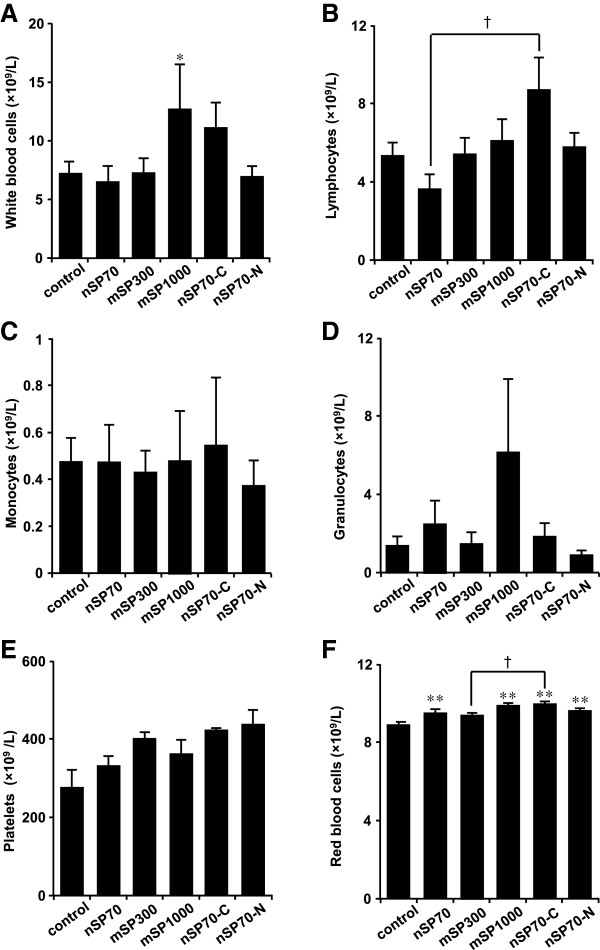
**Hematological analysis after 28-day oral administration of the indicated silica particles.** Hematological analysis of **(A)** white blood cells, **(B)** lymphocytes, **(C)** monocytes, **(D)** granulocytes, **(E)** platelets, and **(F)** red blood cells. Values are expressed as mean ± SE (*n* = 4 to 11). *P < 0.01 and **P < 0.05 compared with the control group; † *P* < 0.05 compared with the nSP70 group, as indicated by Tukey’s test.

Oral exposure of BALB/c mice to nSP70, mSP300, mSP1000, nSP70-C, and nSP70-N at 2.5 mg/mouse for 28 days produced no significant differences in body weight in the treated mice compared with in the control mice (Figure 2A). We observed no significant changes in the plasma levels of ALT (marker of liver function) or BUN (sensitive indicator of kidney damage) after oral administration of the silica particles in treated mice compared with control mice (Figure 2B,C). Although ALT values of silica nanoparticle-treated group are slightly higher than control group, the change of the ALT value was within healthy range (the range was about below 43 U/L) among all the groups. Therefore, we speculate that the level of ALT among all the groups does not effect on liver’s function. Histopathological examination revealed no abnormalities in the liver, kidney, and large intestine (Figure 3) or any other tissue (the brain, lung, spleen, heart, stomach and small intestine; data not shown) in the treated mice. Furthermore, the counts of total monocytes, granulocytes, or platelets in the treated mice were also not significantly different from those in the control mice (Figure 4). Although the white blood cells, lymphocytes, and the red blood cell counts in the treated mice were significantly different from those in the control group, they were within the normal, healthy range or slightly increase (the white blood cells: 6 to 15 × 10^9^/L, lymphocytes: 3.7 to 7.4 × 10^12^/L, red blood cells: 7 to 12 × 10^12^/L) in all groups (Figure 3A,B,F). Taken together, our results indicate that oral administration of nSP70, mSP300, mSP1000, nSP70-C, or nSP70-N for 28 days does not produce any abnormal biological effects, suggesting that the silica nanoparticles tested are safe for use in food production.

## Discussion

In the present study, we attempted to quantify the absorption of silica particles by using ICP-OES to measure the silicon content in the liver and blood after 28-day oral exposure. However, we did not detect the silicon content in the biological tissue with this analytical protocol (data not shown; detection limit of the protocol, 0.1 μg). ICP-OES protocols are regarded to be suitable for measuring silica; however, our attempt to measure ultratrace levels of the silicon content derived from silica particles may have been hindered by interference from nitrogen present in the air. Other groups have also been unable to determine the level of the silicon content derived from with ICP-OES after oral exposure because of interference [11]. We therefore tried to measure the absorption of silica particles through the intestine by means of a combination of the everted gut sac method and ICP-OES. The advantages of this model are that there is a relatively large surface area available for absorption and that a mucus layer is present. The absorption of nSP70-C and nSP70-N were significantly higher than that of the other silica particles, and the absorption of nSP70-C was also significantly higher than that of nSP70 (Figure 1). This indicates that the differences in intestinal absorption of nSP70-C and nSP70-N may be a result of the particles being absorbed via different proteins. Indeed, some groups have suggested that interactions between proteins and nanomaterials play important roles in the biological effects and biodistribution of nanomaterials [12]. In the future, to understand the mechanism of intestinal absorption, we will need to examine the relationships between intestinal absorption and the binding proteins of nSP70, nSP70-C, and nSP70-N. We found that the everted gut sac method was effective for assessing the intestinal absorption of nanomaterials. Although the relationship between models of in vivo absorption and the everted gut sac model of in vitro absorption must be further elucidated, we expect our results to contribute to the development of methods for determining the absorption of silica particles after oral exposure.

We next exposed BALB/c mice to silica particles at an oral dose of 2.5 mg/mouse, which is approximately 10 times the upper safe limit for consumption of silica by adult humans set by the United Kingdom Food Standards Agency’s Expert Group on Vitamins and Minerals (700 mg silica/day) [13]. Since silica nanoparticles are already used as food additives in some food products, for example, coffee creamer (silica nanoparticle content, 1.0 g/kg) and instant soup [1], we consider the dose used in the present study (2.5 mg/mouse) to be high enough for the evaluation of the biological effects of oral administration of the particles. Histopathological, biochemical, and hematological analyses showed no differences between the treated mice and the control mice (Figures 2, 3, and 4). However, another research group has reported that silica nanoparticles (diameter, 30 nm) have toxic effects on the liver when administered orally at a total dose of 140 g/kg over 10 weeks [14], which is a dose approximately 20 times that used in the present study. Therefore, to determine the NOAEL for orally administered silica nanoparticles, their effects on the liver must be precisely elucidated.

Our previous study showed that intravenous or intracutaneous administration of silica nanoparticles caused pregnancy complications [9] and modulation of the immune response [15]. Furthermore, it is possible that intranasally administered silica nanoparticles induce abnormal activation of the coagulation system and modulate the immune response [16,17]. Therefore, in which tissues silica nanoparticles localize after oral exposure, as well as their chronic, reproductive, and immune toxicities, should be determined by using an oral exposure model.

## Conclusions

By using the everted gut sac method coupled with ICP-OES, we examined the intestinal absorption of silica particles and found that surface properties were major determinants of the degree of intestinal absorption. None of the particles tested showed toxic biological effects after 28-day oral administration. Our results suggest that the everted gut sac method coupled with ICP-OES could be one of the effective methods for assessing the absorption of silica particles. The results of this study will be useful for the development of methods for assessing the safety of silica nanoparticles and for the creation of safer nanoparticles.

## Abbreviations

ALT: alanine aminotransferase; BUN: blood urea nitrogen; EDTA: ethylenediaminetetraacetic acid; ICP-OES: inductively coupled plasma optical emission spectrometer.

## Competing interests

The authors declare that they have no competing interests.

## Authors’ contributions

T.Yoshida and YY designed the study. T.Yoshida, HT, KM, TM, and TH performed the experiments. T.Yoshida and YY collected and analyzed the data. T.Yoshida and YY wrote the manuscript. KN, YA, YM, HK, ST, HN, T.Yoshikawa, and KH provided technical support and conceptual advice. YT supervised the project. All authors discussed the results and commented on the manuscript. All authors read and approved the final manuscript.
